# Development and validation of sunlight exposure measurement questionnaire (SEM-Q) for use in adult population residing in Pakistan

**DOI:** 10.1186/1471-2458-12-421

**Published:** 2012-06-08

**Authors:** Quratulain Humayun, Romaina Iqbal, Iqbal Azam, Aysha Habib Khan, Amna Rehana Siddiqui, Naila Baig-Ansari

**Affiliations:** 1 Public Health Solutions Pakistan, House No. 578, Street No. 53, G-9/1, Islamabad, Pakistan; 2 Department of Community Health Sciences, Aga khan University, Stadium road, Karachi, Pakistan; 3 Department of Pathology & Microbiology, Aga Khan University, Stadium road, Karachi, Pakistan; 4 Indus Hospital, Clinical Research Unit, Korangi Crossing, Karachi, Pakistan

**Keywords:** Vitamin D deficiency, Dosimeters, Validation, Correlation, Sunlight exposure

## Abstract

**Background:**

Vitamin D deficiency has been identified as a major public health problem worldwide. Sunlight is the main source of vitamin D and its measurement using dosimeters is expensive and difficult for use in population-based studies. Hence, the aim of this study was to develop and validate questionnaires to assess sunlight exposure in healthy individuals residing in Karachi, Pakistan.

**Methods:**

Two questionnaires with seven important items for sunlight exposure assessment were developed. Fifty four healthy adults were enrolled based on their reported sunlight exposure (high = 17, moderate = 18, low = 19) from Aga Khan University, Karachi. Over four days, study participants were asked to wear a dosimeter between sunrise and sunset and report time spent and activities undertaken in the sun for questionnaire validation. Algorithm for item weightage was created as an average score based on ultraviolet B percentage received. Blood samples were obtained for serum vitamin D.

**Results:**

The mean time (minutes) spent in sun over 4 days (±SD) was 69.5 (±32) for low, 83.5 (±29.7) for moderate and 329 (±115) for high exposure group. The correlation between average time (minutes) spent in sun over 4 days and mean change in absorbance of UV dosimeters for 4 days was 0.60 (p < 0.01). Correlation between average score and vitamin D levels was found to be 0.36 (p = 0.01) for short term questionnaire score, 0.43 (p = 0.01) for long term questionnaire score in summers and 0.48 (p = 0.01) in winters.

**Conclusions:**

The sunlight exposure measurement questionnaires were valid tools for use in large epidemiological studies to quantify sunlight exposure.

## Background

In recent years, exposure to sunlight has been a subject of epidemiological interest both due to its beneficial as well as adverse effects on human health. It is an important etiological factor in the development of skin cancers and sun-related eye problems when exposure is high, or Vitamin D Deficiency (VDD) [1,2]. However, the exposure to solar ultraviolet radiation (UVR) is an essential step for the production of Vitamin D, and also the main source of vitamin D in human body [3,4]. Ultraviolet B (UVB) rays in the solar UVR spectrum produce vitamin D in the human body. UVB rays penetrate uncovered skin and converts cutaneous 7-dehydrocholesterol to pre-vitamin D_3_, which in turn becomes vitamin D_3_[5,6]. The enteral route is not considered a good source of vitamin D as foods of animal origins e.g. fish, beef, eggs, milk, cheese etc., generally lack the required amount of vitamin D unless fortified [7].

VDD used to be considered rare in those parts of the world that had plenty of sunshine all year round but WHO now estimates that globally one billion people have VDD or insufficiency [1,8]. Geographically, Pakistan lies in a region with adequate sunlight throughout the year [9] with UV index ranging between 5 and 11 during different seasons. However, despite its geographical location, the prevalence of VDD is reported to be high, ranging from 20% to 83% [10-12].

Previously sunlight exposure has been measured by dosimeters or by a short sunlight diary. However these tools have certain limitations. The dosimeters are prohibitively expensive, therefore cannot be used in large epidemiological studies and the diaries estimate the duration of exposure to sunlight (time in minutes/day) with adjustment either for none or few covariates that could influence UVB activity, such as use of sunscreens, type of clothing, traveling in sun, working in shady area, sun protection practices and skin tone of the individual etc. A major limitation in the conduct of more research in the area of VDD is the lack of appropriate and inexpensive tools for measuring sunlight exposure, which is an important determinant of vitamin D levels in population based studies. As per our knowledge, no questionnaire is currently available for assessing sunlight exposure in Pakistani population. This study aimed to develop and validate a *Sunlight Exposure Measurement Questionnaire*, for quantification of sunlight exposure in our population.

## Methods

### Study setting and design

This was a validation study and was conducted at the Aga Khan University (AKU) from December 2009 to April 2010, in Karachi, Pakistan. The study population consisted of students, faculty and staff working at AKU, from varied socioeconomic backgrounds living in various parts of Karachi. The study population was selected in three groups according to the perceived sunlight exposure pattern: 1) low sunlight exposure, i.e. those who worked indoors most of the time. This group included faculty and staff; 2) moderate sunlight exposure, this category consisted undergraduate and graduate students; 3) high sunlight exposure, i.e. AKU employees who worked outdoors in the sun most of the day such as security guards, drivers, gardeners and housekeepers. The participants falling in one of the three groups were approached, briefed about the study and those who agreed to participate in the study an informed consent was obtained from them.

### Eligibility criteria

Apparently healthy faculty, staff and students working at AKU were included. Subjects with known skin disorders, immuno-compromised status or a history of using vitamin D injectables or drugs affecting serum vitamin D status in the past one year were not recruited for the study.

### Development of sunlight exposure questionnaires

We developed two pre-coded sunlight exposure measurement questionnaires (SEM-Q) after detailed and thorough literature search. First questionnaire was long term (LT) with reference period of one year and second questionnaire was short term (ST) with reference period of one day (current day) with a grid divided in one hour intervals from sunrise to sunset. Table 1 shows the comparison of the two questionnaires developed for assessing sunlight exposure.

**Table 1:** Comparison of Long Term (LT) and Short Term (ST) Sunlight Exposure Measurement Questionnaires (SEM-Q)

	LT SEM-Q^*^	ST SEM-Q^**^
Reference period	1 year: Previous year summer and winter seasons	1 day: Hourly routine sun exposure recorded for 1 day
Type of questionnaire	Interviewer administered	Self administered (verified)
Domains: Time (minutes) spent outdoors, weather, clothing, use of sunscreen, sun protection practices, use of multivitamin and skin tone.	Typical week or day in previous summers and winters.	Additional domain: Travel in car/bus with windows up. In the form of a grid with time in one hour interval from sunrise to sunset

^*^ Long term sunlight exposure measurement questionnaire;

^**^ Short term sunlight exposure measurement questionnaire; 4 questionnaires were filled out on the four days that dosimeters were worn.

The LT-SEMQ had three elaborate components, the socio-demographic, sun exposure measurement, and skin tone assessment component.

Both the questionnaires were developed in English, translated into Pakistan’s national language, Urdu, and then back-translated into English to check for content validity of the questionnaire.

#### Pre-testing

Pre-testing of the questionnaires was also carried out on 15% of the sample (n = 8) prior to the actual data collection in order to ensure standardization and reliability of the questionnaires. The questionnaires were then revised and finalized on the basis of the pretest results.

#### Factors affecting individual UVR exposure

The personal and atmospheric factors that affect UVB radiation exposure and vitamin D synthesis were included in the questionnaire as domains. These included UVR intensity and exposure duration in the sun [13,14], skin tone of the individual [15], use of sunscreens and other cosmetics [16], other sun protection practices (seeking shade under trees/building [17], clothing [18], hats [19], glass/windows [20], cloud cover [21], and occupational behavior [14,22]. Certain other factors that are considered important determinants of an individual’s exposure to UVR, such as, atmospheric pollution [23], altitude [24], season [25], surface reflection [26], and ozone [27] were either indirectly catered (surface reflection), were not of primary interest (pollution) in the study or were not applicable to our study setting (altitude, season) and hence not included.

### Development of the scoring system for SEM-Q’s

We developed a scoring algorithm for estimation of sunlight exposure (SE) of individuals by taking into consideration all domains that effect SE at an individual level including time (in minutes) of sun exposure and different domains/mediums that protects from sun exposure e.g. use of sunscreen, standing under the tree, clothes worn/body covered. Table 1 also shows the different domains included in the two questionnaires. For short term, the score was developed for SE over one day while for LT SEM-Q, the scoring was created for a typical one week exposure over the previous 1 year for summer and winter seasons separately. The score was developed by giving different weights (ranging between 0 and 1), according to sun exposure (UVB exposure), to all the domains listed in the questionnaire. For example, if the face was not covered, it received 100% UVB, hence a proportion of 1 was given, whereas, if the face was covered or partially covered it received 0% and 50% UVB’s translating into a proportion of 0 and 0.5 respectively. Table 2 shows the different weights given to domain and algorithm established for estimation of sunlight exposure score. The final scoring algorithm was created by multiplying the time (minutes) spent in the sun by the proportions of different domains, as provided by Table 2.

**Table 2:** Algorithm for Estimation of SE Score for Individuals

**Variable/item**	**Percentage given**	**Explanation**
Part of the body exposed (clothing)	1 if exposed	1 = 100% UVB can penetrate.
	0 if not exposed/covered	
	0.5 if partially covered	
Application of sunscreen/cosmetics on different parts of body (SPF)	1 if no use of any products	1 = 100% UVB
	0.08 if sunscreen and SPF 15 and above	0.08 = 8% UVB penetrates i.e. blocks 92%
	0.9 if use of other creams and lotions without knowledge of SPF	0.9 = 90% UVB absorption
Sun protection practices	1 if no protection practices	1 = 100% UVB absorption
	0.4 if used shade of tree/building etc.	0.4 = 40% UVB available for absorption
Weather outdoors	1 if sunny	1 = 100% UVB on a sunny day
	0.5 if cloudy	0.5 = 50% UVB on a cloudy day
	0.75 if sunny/cloudy	0.75 = 75% UVB if light clouds and sunny
Glass windows of car/bus/van	0.1 if glass windows up	0.1 = 10% UVB absorption through glass windows, 90% blocked
	0.4 if windows down	0.4 = 40% UVB available for absorption into skin
Skin tone	Type 1 - 0.8	80% UVB penetration
	Type II - 0.675	67.5% UVB penetration
	Type III - 0.55	55% UVB penetration
	Type IV - 0.425	42.5% UVB penetration
	Type V - 0.3	30% UVB penetration by skin

### Gold standard for sunlight exposure measurement

We used Ultraviolet (UV) dosimeters as the gold standard for validating the questionnaires. Dosimeter measures the amount of UVB ionizing radiation (280–315 nm wavelength) absorbed over a given period of time [24]. The UV dosimeters were purchased and subsequent data analyzed at the University of Southern Queensland (USQ), Queensland Australia. The polysulphone film ultraviolet dosimeters were pinned on the study participant’s clothes as a badge. To remain consistent, all study participants were requested to pin the dosimeter on the upper left side of their chest.

### Study protocol & data collection

Both the questionnaires were administered through a face to face interview. Biochemical assessment was carried out and UV Dosimeter data was collected from each participant. Details of each of these three types of data collection processes are described below.

Participants were administered the LT SEM-Q and at the end of the interview each participant was given four ST SEM-Q and four UV dosimeters, to be used over 4 days. These dosimeters were packed in four individual envelops. Participants were instructed on when and how to wear the dosimeters and to fill the ST SEM-Q at the end of each day that the dosimeter was worn. They were also instructed how to place the used dosimeter back in the respective envelop. The used dosimeters were collected the next day along with the filled ST SEM-Q. The filled ST SEM-Qs were collected after verification, checking for errors and missing information by research assistants for literate participants. The ST SEM-Q was filled the following day for illiterate participants, by the research assistant and same method of interview was employed as that used for verification of ST SEM-Q for literate participants. Interviewer bias and reporting bias were minimized as much as possible by selecting the participants in accordance with the eligibility criteria, by proper training of the research assistants and following the study methodology properly.

**Figure 1:**
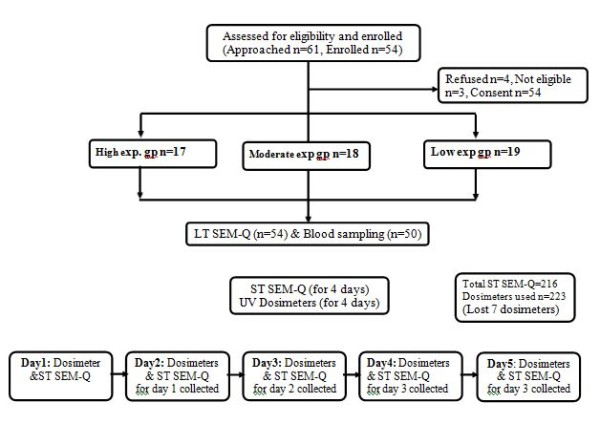
**Recruitment and flow of participants in the study.** Eligible individuals were recruited into the study into three exposure group’s i.e. high, moderate and low exposure, after obtaining informed consent. Participants were administered the LT SEM-Q and at the end of the interview each participant was given four ST SEM-Q and four UV dosimeters, to be used over 4 days. Participants were instructed on when and how to wear the dosimeters and to fill the ST SEM-Q at the end of each day. The used dosimeter and filled ST SEM-Qs was collected the next day after verifying, checking for errors and missing information by research assistants for majority of the participants.

Skin tone of the participants was assessed against shade card by matching shade of the skin on the inner side of the forearm of the participant with the shade on the card. This was a component of LT SEM-Q. Shade card of 20 scales was developed with professional help from experts in designing and printing, to match the skin tones of Asian population. Figure 1 shows the recruitment and flow of participants in the study.

Blood sample, for assessing Vitamin D levels, was obtained from the study participants from December 2009 to April 2010, by trained phlebotomist under sterile conditions. Vitamin D status was determined by measuring serum 25OHD_3_ concentrations by Electrochemiluminescence immunoassay on Elecsys auto analyzer (Roche Diagnostics, USA). For quality control low, medium and high Elecsys Preci Controls were used. The within-run CVs were 5.7%, 5.7%, and 5.4% at concentrations of 25.2, 39.9, and 65.6 ng/ml.

Optimal vitamin D levels were taken as 30–100 ng/ml while levels of 21–29 ng/ml as vitamin D insufficiency and <20 ng/ml as vitamin D deficiency [28]. Vitamin D analysis was done in the laboratory towards the end of the data collection and participants with VDD or insufficiency were informed and educated about sun exposure and vitamin D supplements were provided to the study participants.

### Statistical analysis

For descriptive analysis, means and standard deviations were reported for continuous variables, such as, age, time spent in sun, dosimeter readings; frequencies and percentages were reported for nominal and ordinal variables like VD levels, mean and SD was reported for this variable. For inferential analysis, Kruskal Wallis test was used to detect the difference in sunlight exposure, vitamin D levels and time spent in the sun, among the three sunlight exposure groups. Spearman’s rank correlation and Pearson’s correlation were also carried-out for the association between sun exposure scores & Ultraviolet (UV) dosimeter readings, average scores of SEM-Qs & vitamin D levels and average scores of LT & ST SEM-Qs, respectively. The analysis was carried out using Statistical Package for Social Sciences (SPSS) version 16.0.

### Potential confounders and sources of bias

Potential confounders, such as the outdoor weather, sun protection practices, use of sunscreen and other products, skin tone, and body parts exposed to the sun during the days the dosimeter was worn, were adjusted while creating the scoring for the short term questionnaire.

### Ethical approval

This study protocol was ethically approved by AKU Ethical Review Committee (ERC) on December 4, 2009 (1257-CHS/ERC-09).

## Results

A total of fifty four participants were enrolled in the study. Among the three exposure groups taken, low exposure group accounted for 35.2% of the total sample (n = 19), moderate for 33.2% (n = 18) and high exposure group for 31.5% (n = 17). Male participants constituted 53.7% of the sample. 83.3% of the total participants were literate with 79.6% of the study participants having 10 years or more of formal education. Predominant language spoken was Urdu followed by Punjabi. Muslims were 75.9% of the study participants. Table 3 shows the descriptive analysis of the study participants.

**Table 3:** Demographic Characteristics of Study Participants by Sunlight Exposure Group

**Variables**	**Sunlight exposure groups**			**Total (n = 54)**
	**Low (n = 19)**	**Moderate (n = 18)**	**High (n = 17)**	
Age, yrs [Mean(SD*)]	33 (9)	31 (7)	41 (11)	35 (10)
Sex [n (%)]				
Male	6 (31.6)	7 (38.9)	16 (94.1)	29 (53.7)
Female	13 (68.4)	11 (61.1)	1 (5.8)	25 (46.3)
Literacy rate [n (%)]				
Illiterate	0	0	9 (52.9)	9 (16.7)
Literate	19 (100)	18 (100)	8 (47.1)	45 (83.3)

^*^ Standard deviation.

Serum 25(OH) vitamin D_3_ levels were assessed for 50 study participants. 98% (n = 49) of the participants were either vitamin D deficient or insufficient. The mean vitamin D level (SD) of our study participants was 12 ng/ml (5.9), with levels of 9.8(4.7), 11(4.6) and 17(6.5) for low, moderate and high SE groups respectively. Table 4 shows the distribution of main variables according to the three SE groups.

**Table 4:** Description of Main Variables of Interest

**Variables**	**Sunlight exposure groups**			**Total (n = 54)**
	**Low (n = 19)**	**Moderate (n = 18)**	**High (n = 17)**	
Serum vitamin D (ng/ml) [Mean(SD)]	9.8 (4.7)	11.1 (4.6)	17.0 (6.5)	12.3 (5.9)
PS UV dosimeter (MED) [Median(IQR)]	0.5 (0.1–10.2)	0.8 (0.2–8.2)	3.6 (2.1–11.9)	1.2 (0.1–11.9)
ST SEM-Q*				
Time (minutes) spent outdoors	69.5 (32.0)	83.5 (29.7)	329 (115.7)	95.6 (18.7–487.5)^**^
Adjusted time (minutes) ^***^	43.0 (22.5)	64.9 (21.8)	258 (114.7)	76.3 (12.0–463.8)^**^
LT SEM-Q				
Time (minutes) outdoors in summer [Mean(SD)]	54.5 (30.0)	81(62.7)	331.2 (63.8)	150 (134.9)
Mean (SD) time outdoors in winters	59.7 (32.5)	89.4 (65.0)	310 (85.0)	148 (127.6)

SD=Standard deviation; MED=Minimal erythemal dose; IQR=Inter quartile range;

^*^ Significantly different on Kruskal Wallis test (*P* <0.001); ^**^ Medians (IQR) reported; ^***^ adjusted for weather outdoors, seeking shade & travel in vehicle.

The correlation coefficient between average time (minutes) spent outdoors over the four days for ST SEM-Q, and average readings of UV dosimeter over four days was 0.60 (*P* < 0.01). Spearman’s rank correlation between average score of ST SEM-Q and serum vitamin D levels was found to be 0.36 (*P* = 0.01), while Pearson’s correlation between serum vitamin D levels and average LT SEM-Q score in summers was 0.43 (*P* = 0.01) and 0.48 (*P* = 0.01) in winters. Table 5 shows the correlations among the main variables of interest.

**Table 5:** Correlations between Questionnaires and Dosimeter Readings, and Serum Vitamin D Levels

		**Spearman’s Rank Correlation**	***P* -value**
ST SEM-Q			
Average time (minutes) spent in sun	UV Dosimeter readings (MED)	0.601	< 0.001
Adjusted-average time (minutes)^*^	UV Dosimeter readings (MED)	0.534	<0.001
ST SEM-Q as a score	Serum vitamin D levels	0.363	0.01
LT SEM-Q			
Time (minutes) spent in sun in summers	UV Dosimeter readings (MED)	0.582	0.01
Time (minutes) spent in sun in winters	UV Dosimeter readings (MED)	0.613	0.01
Score of sun exposure in summers (per day)	Serum vitamin D levels	0.429^**^	0.01
Score of sun exposure in Winters (per day)	Serum vitamin D levels	0.483^**^	0.01

ST SEM-Q=Short term sunlight exposure measurement questionnaire; MED=minimual erythemal dose, LT SEM-Q= Long term sunlight exposure measurement questionnaire.

^*^ adjusted for sun protection practices, weather and travelling in vehicle; ^**^ Pearson’s Correlation reported.

Correlation between LT and ST questionnaires was assessed. It was found that the correlation coefficient between average time (minutes) spent outdoors as captured by LT and ST was 0.85 (*P* < 0.01). Similarly, the correlation coefficient between the scores for LT and ST SEM-Q was observed to be 0.82 (*P* < 0.01).

## Discussion

We found a good correlation between our ST SEM-Q and dosimeter readings (r_s_ = 0.60 (p < 0.01)) and fair correlation of 0.36 (*P* = 0.01) between ST SEM-Q and serum vitamin D levels. Similarly, a good correlation of 0.58 (*P* = 0.01) and 0.60 (*P* = 0.01) was observed between dosimeter readings and LT SEM-Q summer and winters respectively. The correlation between vitamin D levels and LT SEM-Q score for previous year summer and winter season was found to be 0.43 (*P* = 0.01) and 0.48 (*P* = 0.01) respectively.

Through recent literature search, this study is the first of its kind in South Asian region to develop a questionnaire to assess long and short-term sunlight exposure and validate it against a gold standard objective measure using dosimeter badges. It is also the first study to assess the correlation between the SEM-Q and serum vitamin D levels in Pakistan. Our research strength is that we developed the questionnaire based on the factors that are of importance in a non-western society. It is also a culturally sensitive and acceptable questionnaire in our setting.

The correlation we found between our ST-SEMQ questionnaire and the dosimeter readings (r_s_ = 0.60; *P* < 0.01) were higher than the Australian pilot study, which compared the objective measurement and self-reported ultraviolet radiation exposure during outdoor activities among children and mother pairs and life guards (r = 0.32) [29]. The main Australian study later showed fair to good correlations between the sunlight diaries and dosimeters for lifeguards (r = 0.38–0.57), parents (r = 0.28–0.29) and children (r = 0.18–0.34) and these findings were still lower than our study findings [30]. Similarly, our results were higher than previously published data on mothers and children [31], school children [32] and volunteers recruited from recreational organizations [25]. A possible reason for better correlation of our questionnaire could be the comprehensive nature of the questionnaire and inclusion of items relevant for our population. Moreover, our study population consisted of adults only, working for a university, which could be a reason for better response. Also, most of the studies done previously had included factors in relation to skin cancer development and had taken the time of day when the ultraviolet radiations are the most intense (11 am to 3 pm) as reference period. We included larger time of sun exposure, which most of the other studies had not taken into consideration.

The results of our study were comparable to some of the other studies that compared personal UVR exposures using PS dosimeters and diary or questionnaire entries among adult volunteers (r = 0.69) [33] and school children (r = 0.68) [34].

Our correlation between serum Vitamin D and average score for ST SEM-Q was found to be 0.36 (*P* = 0.016), 0.43 (*P* = 0.01) for LT SEM-Q score in summers and 0.48 (*P* = 0.01) for LT SEM-Q score in winters. We did not come across any study that developed scoring for sun exposure questionnaires to assess the correlation between the scores and vitamin D levels hence we are unable to compare our results with any other study. One study that did measure Vitamin D levels and related it to UVR behavior, correlated the mean daily hours measured by dosimeter in September and Feburary (r = 0.64, *P* = 0.001) and (r = 0.53; *P* = 0.007) [35], however, the study did not look into correlation between Vitamin D and scores for sunlight diaries or questionnaires.

There were a few limitations to our study. The fair correlations of 0.34, 0.43 and 0.48 observed between the serum vitamin D levels and scores for SEM-Qs were probably because of the smaller sample size and hence the inability to find a variation in terms of vitamin D levels, as majority of study sample was vitamin D insufficient or deficient. Even though our estimated sample size was larger (n = 203), due to budget constraints and time limitations, the study was conducted on approximately 25% of the sample size. We expect that with the larger sample, we would have achieved a larger variation in terms of vitamin D levels and probably a better correlation with the computed score. However, the sample size of 54 with a post hoc power of 83% was sufficient for meeting the main objective of our study.

We developed and validated two questionnaires i.e. ST and LT SEM-Q, in order to measure and assess both short term sunlight exposure and long term SE over previous 1 year period. Our study assessed sunlight exposure in context with Vitamin D levels, unlike the recent other studies that have assessed sunlight exposure in relation to skin cancer [36]. Both the questionnaires showed good correlation with dosimteres and fair correlations with serum vitamin D levels and, therefore, we can say that both the questionnaires can be used to assess the SE in adults. However, LT SEM-Q might be preferred due to convenience of its use and reflection of a longer duration of sun light exposure inadequacy which may be more relevant for epidemiological work.

The UV dosimeters, although gold standard for measuring UVR, have a few limitations which makes the questionnaires preferred source for assessing sunlight exposure. UV dosimeters are expensive tools and are not readily available in developing countries and hence need to be imported for use. Secondly, the calibration and reading of dosimeters is a complex process and require sensitive equipment and expertise, which is not easily available especially in our settings. Lastly, the dosimeters measure the current exposure to sunlight and do not take into account the long term exposure to sunlight, which is an important factor for vitamin D levels in the body.

## Conclusions

The main outcome of this study was development and validation of a culturally-appropriate and inexpensive tool for assessing sunlight exposure. We found a high correlation between self-reported sun exposure using our tool and the objective measure of sun exposure using the dosimeters and, therefore, SEM-Qs can be used in population based studies for assessing SE. This has great epidemiological value for work in the related vitamin D sunlight exposure measurement and for assessing SE in relation with other disease outcomes such as skin cancers.

Furthermore, this is the first study in our region for assessing the exposure to sunlight through a questionnaire, and which incorporates methods for scoring based on the factors that affect the UVB rays absorption in the body, factors that may directly affect the amount of pre-vitamin D_3_ synthesis in the skin.

## Competing interests

The authors declare that they have no competing interests.

## Authors’ contributions

QH participated in study design, tool and methods development and carried out data collection, analysis and write up. RI conceived the idea and supervised every stage of the study. ARS participated in materials and methods development and in technical review of the project. IA participated in the statistical analysis and tool development. AHK and NBA helped to draft the manuscript and gave important inputs. All authors reviewed the manuscript and approved the final version.

## Pre-publication history

The pre-publication history for this paper can be accessed here:

http://www.biomedcentral.com/1471-2458/12/421/prepub
